# Qualitative description – the poor cousin of health research?

**DOI:** 10.1186/1471-2288-9-52

**Published:** 2009-07-16

**Authors:** Mette Asbjoern Neergaard, Frede Olesen, Rikke Sand Andersen, Jens Sondergaard

**Affiliations:** 1 The Research Unit for General Practice, Aarhus University, Bartholins Allé 2, DK-8000 Aarhus, Denmark; 2 Department and Research Unit of General Practice, University of Southern Denmark, J.B. Winsløws Vej 9, DK-5000 Odense C, Denmark

## Abstract

**Background:**

The knowledge and use of qualitative description as a qualitative research approach in health services research is limited.

The aim of this article is to discuss the potential benefits of a qualitative descriptive approach, to identify its strengths and weaknesses and to provide examples of use.

**Discussion:**

Qualitative description is a useful qualitative method in much medical research if you keep the limitations of the approach in mind. It is especially relevant in mixed method research, in questionnaire development and in research projects aiming to gain firsthand knowledge of patients', relatives' or professionals' experiences with a particular topic. Another great advantage of the method is that it is suitable if time or resources are limited.

**Summary:**

As a consequence of the growth in qualitative research in the health sciences, researchers sometimes feel obliged to designate their work as phenomenology, grounded theory, ethnography or a narrative study when in fact it is not. Qualitative description might be a useful alternative approach to consider.

## Background

The growth in qualitative health sciences research has led to the introduction of a vast array of qualitative methodologies, resulting in what Margarete Sandelowski has called 'methodological acrobatics' [1], meaning that researchers sometimes feel obliged to designate their work as phenomenology, grounded theory, ethnography or a narrative study when in fact it is not. This may result in 'posturing' and does not make any methodological or theoretical contributions. Furthermore, it may neglect the benefits of an alternative approach, namely qualitative description [1].

The knowledge and use of qualitative description (QD) as a qualitative research approach in health research is limited and is often criticised for being too simple and lacking rigour [1,2]. However, proper use of the method can provide useful data tailoring clinical interventions, scales, needs assessments and questionnaires in mixed method studies or in relation to small independent research projects [3].

The aim of this article is to discuss the potential benefits of a QD approach, to identify its strengths and weaknesses and to provide examples of use.

## Discussion

### Qualitative and Quantitative Research

QD follows the tradition of qualitative research, i.e. an empirical method of investigation aiming to describe the informant's perception and experience of the world and its phenomena. Qualitative research is well suited for *"why"*, "*how" *and "*what" *questions about human behaviour, motives, views and barriers. Thus, with its mainly inductive approach qualitative research is suitable for problem identification, hypothesis generation, theory formation and concept development [4].

With their deductive approach quantitative methods are well suited for *"when"*, *"how much" *and *"how many" *questions and are therefore suitable for problem quantification and testing of theories, interventions and new treatments. It seems evident that qualitative and quantitative methods can supplement each other in analysing a research topic from different perspectives [4].

### How does Qualitative Description differ from other qualitative methods?

Qualitative researchers in health sciences have diverse backgrounds; most of them are inspired by phenomenological and hermeneutical traditions, and their approach is mainly theory-driven [5], however QD is founded in existing knowledge, thoughtful linkages to the work of others in the field and clinical experience of the research-group. The various qualitative approaches focus on various phenomena and thus produce different results. Both description and interpretation are legitimate but they are tied to different conditions and interests [6].

QD differs from other qualitative methods in several ways. Firstly, in terms of analysis, the aim of QD is neither thick description (ethnography), theory development (grounded theory) nor interpretative meaning of an experience (phenomenology), but a rich, straight description of an experience or an event. This means that in the analytical process and presentation of data, researchers using QD stay closer to the data. Whereas other qualitative approaches often aim to develop concepts and analyse data in a reflective or interpretive interplay with existing theories, the final product of QD is a description of informants' experiences in a language similar to the informants' own language [1,3]. A central discussion related to QD is, however, whether 'pure description' in positivistic terms is possible. In line with Sandelowski, one could say that QD involves low-inference interpretation meaning that even though description is the aim of QD interpretation is always present. Hence, descriptions depend on the perceptions, inclinations, sensitivities and sensibilities of the describer [6].

Secondly, the interview guide used in QD is slightly more structured than in other qualitative methods although it is still modified and transformed as themes emerge during the analysis. The interview guide is typically based on expert knowledge to focus on areas that are either poorly understood in a health care context and/or potentially amenable to intervention.

On the other hand QD, as any other qualitative approach, may be inspired of other approaches and have textures from either phenomenological, grounded theory, ethnographic or narrative approaches [1]. These overtones in QD can be confusing to the untrained researcher and exactly lead one to claim using methods they are in fact not using.

Furthermore, QD should not be mistaken for interpretive description [3,7] or pattern analysis [3]. The main difference between QD and interpretive description lies in the data analysis, where interpretative description goes beyond mere description and aims to provide an in-depth conceptual description and understanding of a phenomenon, and QD stays closer to the data obtained. The analytic procedures in interpretive description capitalize on such processes as synthesizing, theorizing and recontextualizing rather than simply sorting and coding [7,8]. Pattern analysis seeks to describe patterns in data based on specific factors such as demographics, structural issues or socioeconomic status.

### Design of a Qualitative Description study

The design features are only concisely summarised in this article. For a more thorough exposition we recommend the paper of Sandelowski [1]. The design issues of QD proposed by Sandelowski are seen in Table 1[1]. However, these issues are not exhaustive or exclusive in any way, since the way to do QD remains flexible.

**Table 1:** QD design issues as proposed by Sandelowski [1]

Qualitative Description Design Issues	
**Design issues**	**Design specifics**

Philosophy	Pragmatic approach Overtones of other qualitative approaches (phenomenology, grounded theory, ethnography or a narrative study)
Sample	Purposeful sampling Maximum variation sampling is especially pertinent
Data collection	Minimally-to-moderately structured open-ended interviews with individuals or focus groups Researchers are interested in the Who, What, Where and Why of the experience Observation of specific occurrences Review of documents or other pertinent materials
Analysis	Qualitative content analysis using modifiable coding systems that correspond to the data collected When appropriate "Quasi-statistical" analysis methods are added using numbers to summarize data with descriptive statistics Stay close to the data – low level interpretation (if using qualitative software such as NVivo, the use of "in vivo coding" procedures works well here) Goal of the analysis strategy is to understand the latent variable (useful for concept clarification and instrument development)
Outcomes	Straight description of the data organized in a way that "fits" the data (chronologically by topic, by relevance etc.)

#### Theoretical framework

As mentioned above QD is probably the least theoretical of the qualitative approaches. QD is founded in existing knowledge, thoughtful linkages to the work of others in the field and clinical experience of the research-group

#### Sampling

Any of the purposeful sampling techniques may be used in QD studies. Especially maximum variation sampling seems useful to get a broad insight into a subject.

#### Data collection

Usually semi-structured interviews with open-ended questions are used in QD. It can be individual and/or focus group interviews. Especially focus group interviews seem pertinent to get a broad insight into a subject.

#### Data analysis

The strategy of content analysis is used in QD and is common to many qualitative methods and is described in more detail in Table 2[9].

**Table 2:** Analytic strategies as proposed by Miles et al [9]

Six analytic strategies in QD
a. Coding of data from notes, observations or interviews
b. Recording insights and reflections on the data
c. Sorting through the data to identify similar phrases, patterns, themes, sequences and important features
d. Looking for commonalities and differences among the data and extracting them for further consideration and analysis
e. Gradually deciding on a small group or generalizations that hold true for the data
f. Examining these generalizations in the light of existing knowledge

In some QD studies 'Quasi-statistical' analysis methods are added using numbers to summarize data with descriptive statistics. In this way descriptive or interpretive validity is sought since most people would agree to the accurate accounting of events or meanings [1]. Reporting frequency of data bits is indeed a description and stays close to the data. However, 'Quasi-statistical' analysis methods do not stand alone as the result in a QD study and is merely a supplement to the content analysis.

#### Reporting the results

When reporting results in QD one stays close to the data and describes informants' experiences in a language similar to the informants' own language.

### Strengths and weaknesses of Qualitative Description

All methods have limitations. QD is often criticised for being neither clear nor theory-based [2]. However, this criticism is only justified if QD is used for the wrong purposes.

QD should be the method of choice only when a description of a phenomenon is desired. In terms of analysis this imposes certain limitations, as the low-inference approach reduces the ability to speak in general terms. When data are neither generated nor interpreted on the basis of existing theories or knowledge of a given subject, only a descriptive summary can rightly be given. However, such summaries may yield the working hypotheses or key categories for future theory-based research [1].

Furthermore, QD has been criticised for its lack of rigour and for being flawed, when it comes to judging its credibility. However, it is possible to establish both rigour and credibility in QD. Milne and Oberlee talk about enhancing rigour in QD by focusing on the strategies seen in Table 3) [2]. All these criteria are important and overall in line with Guba and Lincoln's lasting argument that qualitative research (or naturalistic inquiry – as they call it) needs other criteria for meeting credibility than more positivistic approaches [10].

**Table 3:** Strategies to enhance rigour in QD as proposed by Milne et al [2]

Strategies to enhance rigour in QD	
**Strategy**	**Techniques**

Authenticity	The informants are free to speak
	Purposeful, flexible sampling
	Participant-driven data collection
	The informants' voices are heard
	Promoting richness rather than superficiality of data
	Conducting focus group interviews to diminish the role of the researcher
	Informants' perceptions are accurately represented
	Accurate transcription
	Content analysis (ensuring data-driven coding and categorizing)
Credibility	Capturing and portraying a truly insider perspective
Criticality	Reflection on the critical appraisal applied to every research decision
Integrity	Reflecting on researcher bias
	Dual role (clinician/researcher/interviewer) during the interview
	Dual role in the process of analysing
	Informants' validations/member checking
	Peer review/researcher triangulation

When analysing QD data, no theoretical strings are attached. This is positive in that the analysis stays close to the data and the informants' points of view. However, it may make the analytical process somewhat subjective as descriptions will always depend on the researcher's perceptions, inclinations, sensitivities, and sensibilities [1]. It is therefore important to reduce the subjective element by involving a group of researchers in the analytic process. The most important criteria to meet when using QD is therefore that of integrity [2] or neutrality [10].

As mentioned above, QD ties nicely in with quantitative data and is useful for mixed method inquiries since it is very suitable for intervention development or refinement, conceptual clarification underlying scale development and needs assessments, especially in vulnerable populations [3]. This seems to be a relevant approach in health services research where the patients' perspectives and evaluations are a quality goal in itself, since QD presents the facts from exactly the informants' points of view. Furthermore, it is a way of gaining a first insight into the informants' views of a particular, narrow topic.

### Examples of QD applied in health services research

We applied QD in two studies. The first was a mixed method PhD study entitled *"Palliative home care for cancer patients in Denmark – with a particular focus on the primary care sector, GPs and community nurses"*. The aim was to investigate how palliative care was provided, what factors were involved in successful and unsuccessful palliative pathways, and what could be done to make improvements. Furthermore, we wanted to identify the statistically significant factors that seemed to be present in the successful and unsuccessful palliative pathways.

A combined QD and questionnaire study seemed very appropriate to achieve the aim. The QD-study yielded appropriate results for the development of the questionnaires, the planning of future palliative home care and research and, furthermore, enabled us to meet the deadline of the project (Neergaard MA et al. Shared care in basic level palliative home care – organizational and interpersonal challenges. A qualitative group interview study. Submitted to Fam Pract 2009) [11].

The other study was a small independent research project carried out by a General Practitioner (GP) as a mandatory part of the Nordic Specialist Course in Palliative Medicine. The GP had no research experience prior to carrying out the project. The GP was interested in how it would influence the spouse's experience of the palliative course of disease to be actively involved (administering and being in charge of oral or subcutaneous medication or assisting with the patient's personal hygiene) [12]. QD proved to be a successful method in more than one way. It was very easy to explain the step by step method to the GP as the project progressed, and it was easy to maintain focus on the specific topic of the study. Furthermore, the deadline of the project was again easily met in spite of the qualitative research approach.

### Perspectives

QD is a useful method for many research questions in health care because it can help to focus on the experiences of patients, relatives and professionals and their views on the patient-professional interaction and the organisation of the health care system. The method can be learned and used by all medical professions without a formal theoretical education in qualitative research methods, however, it obviously needs to be supervised by a trained researcher.

It is always important to carefully consider which method to use prior to initiating the project. QD is a descriptive approach, and whenever a more in-depth theory-based analysis of a subject is needed, QD is not the right method. The strengths of QD are, however, evident in relation to mixed method research because a QD study conducted prior to the development of a questionnaire or an intervention can give very important, useful information. In relation to our mixed method study the QD study among other issues, revealed that problems with shared care were much larger in palliative home care than we had expected prior to our study. This gave us the opportunity to add this as an important theme in our questionnaire. Furthermore, QD turned out to be an appropriate qualitative method for a small interview study where we wanted to gain preliminary insight into a specific topic. Clinicians who want to carry out a small research project are often presented with a quantitative project, but by using QD for this assignment they will have an opportunity to stay close to patients' or families' views. Hence, using QD may also prove to be a useful method for recruiting and retaining clinicians in research.

It has been debated whether QD is a categorical or non-categorical alternative to other research methods. Sandelowski argues that it is a categorical alternative since it is an existing method, yet relatively unacknowledged, as opposed to being a new adaption of grounded theory, phenomenology or ethnography [1]. But no matter what point of view one has according to this debate, qualitative description is a useful qualitative method in much medical research if you keep the limitations of the approach in mind. It is especially relevant in mixed method research, in questionnaire development and in research projects aiming to gain firsthand knowledge of patients', relatives' or professionals' experiences with a particular topic. Another great advantage of the method is that it is suitable if time or resources are limited.

## Summary

The growth in qualitative research in the health sciences has led to the introduction of a vast array of qualitative methodologies. As a consequence, researchers sometimes feel obliged to designate their work as phenomenology, grounded theory, ethnography or a narrative study when in fact it is not. A useful alternative approach, qualitative description, seems to have fallen into oblivion.

## Abbreviations

QD: Qualitative description; GP: General practitioner.

## Competing interests

The authors declare that they have no competing interests.

## Authors' contributions

MAN conceived the idea and drafted the manuscript.

All authors read, revised and approved the final manuscript.

## Pre-publication history

The pre-publication history for this paper can be accessed here:

http://www.biomedcentral.com/1471-2288/9/52/prepub
